# Prognostic role of short physical performance battery in elderly hospitalized atrial fibrillation patients

**DOI:** 10.1007/s11739-025-03958-8

**Published:** 2025-05-16

**Authors:** Giuseppe Armentaro, Danilo Menichelli, Daniele Pastori, Federica Fazio, Caterina Benincasa, Marilisa Panza, Ilaria Gareri, Carlo Alberto Pastura, Marcello Divino, Giandomenico Severini, Alberto Castagna, Pasquale Pignatelli, Francesco Andreozzi, Giovanni Ruotolo, Angela Sciacqua

**Affiliations:** 1 Geriatrics Division, “Renato Dulbecco” University Hospital of Catanzaro, 88100 Catanzaro, Italy; 2 Department of Clinical Internal, Anesthesiological and Cardiovascular Sciences, Sapienza University of Rome, 00161 Rome, Italy; 3 Department of Medical and Surgical Sciences, University Magna Græcia of Catanzaro, 88100 Catanzaro, Italy; 4 Primary Care Department, Azienda Sanitaria Provinciale Catanzaro, 88068 Soverato, Italy; 5 Geriatrics Unit, “Renato Dulbecco” University Hospital of Catanzaro, 88100 Catanzaro, Italy

**Keywords:** Atrial Fibrillation, Elderly, DOACs, Short physical performance battery

## Abstract

Atrial Fibrillation (AF) is the most common supraventricular arrhythmia, especially in elderly, in whom may contribute to physical impairment. The Short Physical Performance Battery (SPPB) is particularly important for assessing the overall physical functionality but the prognostic role of functional limitation, assessed with the SPPB, in elderly hospitalized AF patients is unknown. The objective of this study is to assess the potential impact of functional disability, evaluated as SPPB < 8 points, on the prognosis of elderly patients with AF who are hospitalized. In this retrospective analysis, 1004 elderly patients were enrolled, of whom 620 women, with a mean age of 84 ± 7.1 years. The occurrence of cardiovascular events CVEs (non-fatal ischemic stroke, nonfatal myocardial infarction, cardiac revascularization or coronary bypass surgery, and cardiovascular death), total mortality occurrence were evaluated during a median follow-up of 3.2 years. In patients with SPPB ≥ 8 pt, the observed CVEs incidence was 3.8 events/100 patient-year compared to 12.4 events/100 patient-year in those with SPPB < 8 (p < 0.0001). The multivariate analysis model confirmed that SPPB < 8 was associated with an increased risk of CVEs by a factor of 2.78. In addition, the presence of pathological geriatric depression scale, previous use of Vitamin K Antagonists, were associated with an increased risk of CVEs in study participants. In contrast, statin therapy, male sex and renin–angiotensin–aldosterone system inhibitors are associated with a reduced risk of CVEs during follow-up. Hospitalized patients with AF that exhibits pathological SPPB score show a higher risk of CVEs and total mortality.

**Supplementary Information:**

The online version contains supplementary material available at 10.1007/s11739-025-03958-8.

## Background

Atrial fibrillation (AF) is the most common supraventricular arrhythmia in elderly and is destined to rise as it is directly related to ageing population [1]. AF increases the risk of ischemic stroke, myocardial infarction, heart failure (HF), sudden death and overall cardiovascular mortality and hospitalization [2, 3]. Oral anticoagulant therapy, especially direct oral anticoagulants (DOACs) which are preferred over Vitamin-K antagonists (VKAs), represent the cornerstone for prevention of stroke and systemic embolism in patients with AF due to their better safety profile [4–7].

In addition to increasing the risk of stroke, AF is associated with a series of non-cardiovascular comorbidities, that worsening symptoms, quality of life, and determining an important functional limitation, therefore complicating its management [6, 7]. However, there are no data regarding the prognostic role of functional limitation in elderly hospitalized patients with non-valvular AF. The aim of the present study was to examine the long-term prognostic role of Short physical performance battery (SPPB) in predicting cardiovascular events (CVES) in elderly hospitalized patients with AF treated with DOACs.

## Methods

In this retrospective study, 1004 patients were recruited between 2012 and 2022 at the two Geriatrics Units of the “Renato Dulbecco” University Hospital in Catanzaro. The inclusion criteria were: (I) age ≥ 65 years; (II) previous diagnosis of AF; (III) anticoagulant therapy with DOACs. The exclusion criteria were: (I) AF diagnosed for the first time at the time of enrollment; (II) chronic kidney disease in stage IV K- DOQI (eGFR < 30 ml/min/1.73 m^2^) or dialysis; (III) liver cirrhosis (Child–Pugh Class C); (IV) previous diagnosis of severe psychiatric disorders; (V) autoimmune diseases; (VI) previous diagnosis of dementia; (VII) severe depressive disorders or on therapy versus this disease. Diagnosis and pattern of AF, EHRA score, CHA_2_DS_2_VASc, and HAS-BLED scores were assessed according to 2020 European Society of Cardiology (ESC) guidelines [1].

Anemia was defined by hemoglobin levels < 13 g/dl for males and < 12 g/dl for females. Major and clinically relevant non major bleedings were defined according to International Society of Thrombosis and Hemostasis criteria [6], [8]. At the time of enrollment, a thorough medical history was collected for all patients, assessing comorbidities, cardiovascular risk factors, pharmacological therapy; and a complete physical examination was performed. A 12-lead ECG was performed using a Philips Page Writer T10 electrocardiograph. All laboratory measurements were performed after at least 12 h of fasting. Blood glucose was determined using the glucose oxidase method (Beckman Glucose Analyzer II, Beckman Instruments). Blood levels of total cholesterol, low-density lipoprotein cholesterol (LDL), high-density lipoprotein cholesterol (HDL), triglycerides were analyzed using enzymatic methods (Roche Diagnostics GmbH, Mannheim, Germany), insulin was quantified using a chemiluminescent assay (Immulite, Siemens, Italy). Creatinine levels were measured using the Jaffé method. The estimated glomerular filtration rate (eGFR) was based on the CKD-EPI equation (Chronic Kidney Disease Epidemiology Collaboration) [9], and creatinine clearance (CrCl) was calculated using the Cockcroft-Gault formula [10]. Chronic kidney disease (CKD) was defined as Chronic Kidney Disease Epidemiology Collaboration group (CKD-EPI) formula value < 60 ml/min/1.73 m^2^. Serum uric acid (UA) levels were assessed using the URICASE/POD method (Boehringer Mannheim, Mannheim, Germany). All participants underwent a comprehensive geriatric assessment (CGA) using the following assessment scales: Mini Mental State examination (MMSE) [11], Montreal Cognitive Assessment (MoCA) [12], Geriatric Depression Scale (GDS) [13], Activity of Daily Living (ADL) [14], Instrumental Activity of Daily Living (IADL) [15], Short Physical Performance Battery (SPPB) [16].

### Clinical endpoints

The clinical evaluation, CGA, laboratory tests, and ECG assessment were conducted to evaluate the possible association between the different study variables and the incidence of cardiovascular events (CVEs), non-cardiovascular mortality, and total mortality were evaluated during the follow-up. CVEs included non-fatal ischemic stroke, non-fatal myocardial infarction (MI), and cardiovascular death. The diagnosis of myocardial infarction was made according to the universal definition proposed jointly by the ESC/ACCF/AHA/WHF [17]. If a patient died within four weeks after a stroke or an MI, the event was classified as fatal. Cardiovascular death includes sudden death, progressive heart failure, and death related to surgical or percutaneous revascularization procedures. The diagnosis of ischemic stroke was determined based on clinical manifestations and confirmed by radiological findings [18]. Data on CVEs were collected during follow-up. When an event occurred, a standardized form was completed by the examiners. Details of each event were recorded, including death certificates, hospital discharge letters, copies of hospitalization records, and other clinical documentation obtained from patients. The assessment of cardiovascular events was performed by a committee composed of physicians who did not participate in the recruitment. Each committee member independently and blindly evaluated and adjudicated the events.

### Statistical analysis

**Fig. 1:**
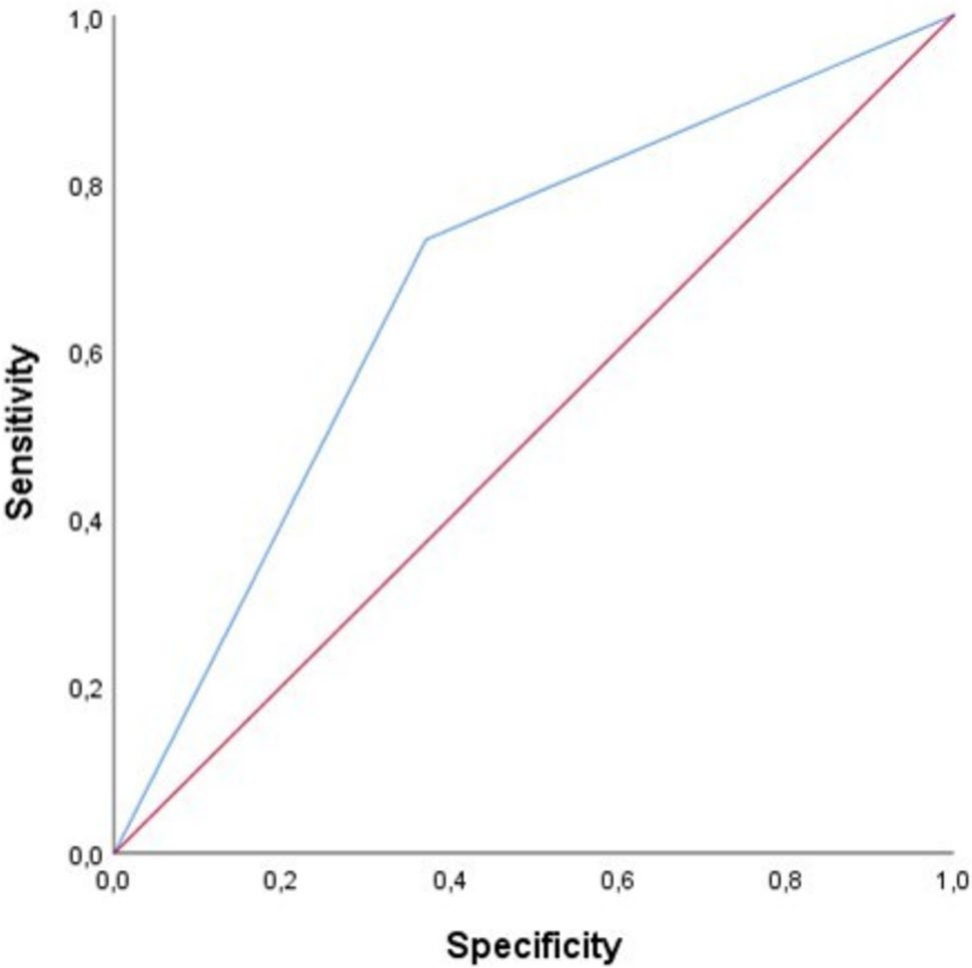
ROC curve of SPPB as dichotomous value as a predictor of CVES occurrence. *ROC* receiver operating characteristic; *SPPB* Short physical performance battery, *CVES* cardiovascular events

Data were expressed as mean ± standard deviation (SD) for normally distributed continuous variables, as median and interquartile range (IQR) for non-normally distributed data, and as number and percentage for binary categorical variables. The overall population was divided into two groups based on the SPPB score (cut-off < 8 points) as the main parameter of lower limb motor function. Comparisons between the two groups were performed using the Student's t-test for normally distributed continuous variables, the Mann–Whitney U test for non-normally distributed continuous variables, and the chi-square test for categorical variables. The accuracy of the SPPB parameter as a predictor of CVEs onset, as a dichotomous variable, was evaluated by constructing a receiver operating characteristic (ROC) curve, as shown in Fig. 1. The area under the curve (AUC) described how to measure the SPPB value associated with the onset of events. The incidence of events was calculated as the number of events per 100 patient-years. Cox proportional hazards regression analysis was used to estimate risk factors for CVEs, correcting the analysis for potential confounders. A univariate Cox regression model was performed on the incidence of CVEs. For each variable, the Hazard Ratio (HR) with the 95% confidence interval (CI 95%) was calculated. Variables that were correlated with the onset of CVEs were included in a multivariate Cox regression model to calculate the HR for independent predictors associated with the incidence of CVEs. All values with p ≤ 0.05 were considered statistically significant. All analyses were performed using the SPSS 20.0 statistical software for Windows.

## Results

The study population consisted of 1,004 elderly patients with AF on DOACs therapy, including 620 women and 384 men with a mean age of 84.0 ± 7.1 years. Among them, 541 had an SPPB score of ≥ 8 points (first group), and the remaining 463 patients had an SPPB score of < 8 points (second group). The mean follow-up duration was 3.2 ± 1.7 years. Table 1 shows the prevalence of comorbidities and therapies of the entire population stratified according to the SPPB score. The group with pathological SPPB had a significantly higher age than the other group (87.4 ± 5.3 vs 81.1 ± 7.3 years; p < 0.0001) and a higher prevalence of women (68.9% vs 55.6%; p < 0.0001). Of interest, 46.5% of the study population had previously been treated with vitamin K antagonists (VKAs) before starting DOACs therapy; 7% were on concomitant antiplatelet therapy.

**Table 1:** Comorbidities, Treatments, laboratory and clinical variables stratified according to SPPB Score

	All population (n. 1004)	SPPB ≥ 8pt (n. 541)	SPPB < 8 pt (n. 463)		p
Comorbidities					
Sex, f/m (%)	620/384 (62/38)	301/240 (55.6/44.4)	319/144 (68.9/31.1)		** < 0.0001**
Age, years (%)	84 ± 7.1	81.1 ± 7.3	87.4 ± 5.3		** < 0.0001**
Smokers, n (%)	243 (24.2)	150 (27.7)	93 (20)		**0.004**
Alcool, n (%)	96 (19.5)	48 (8.9)	48 (10.4)		0.422
Arterial Hypertension, n (%)	804 (80)	421 (77.8)	383 (82.7)		0.052
T2DM, n (%)	335 (33.3)	174 (32.3)	161 (34.8)		0.381
NAFLD, n (%)	85 (8.4)	47 (8.7)	38 (8.2)		0.785
Dyslipidemia, n (%)	232 (23.1)	130 (24)	102 (22)		0.453
RF/COPD, n (%)	341 (33.9)	201 (37.1)	140 (30.2)		**0.021**
IHD, n (%)	196 (19.5)	114 (21)	82 (17.7)		0.180
HF, n (%)	412 (41)	218 (40.2)	194 (41.9)		0.606
CHA2DS2 VASc, pt	4.9 ± 1.4	4.7 ± 1.4	5.2 ± 1.5		** < 0.0001**
HAS-BLED, pt	2.4 ± 0.8	2.4 ± 0.7	2.8 ± 0.8		**0.007**
Pharmacotherapies					
VKAs pre-DOACs, n (%)	467 (46.5)	248 (45.8)	219 (47.3)		0.644
Anti-PLTs pre-DOACs, n (%)	150 (14.9)	69 (12.8)	81 (17.5)		**0.036**
Anti-PLTs, n (%)	71 (7)	35 (6.5)	36 (7.8)		0.421
Nitrates, n (%)	59 (5.8)	29 (5.4)	30 (6.5)		0.452
RAASi, n (%)	680 (67.7)	369 (68.2)	319 (68.9)		0.444
β-bloccanti, n (%)	782 (77.8)	416 (76.9)	366 (79)		0.412
Digitalis, n (%)	96 (9.5)	55 (10.2)	41 (8.9)		0.481
CCBs, n (%)	82 (8.1)	53 (9.9)	29 (6.3)		**0.041**
OADs, n (%)	162 (16.1)	76 (14)	86 (18.6)		0.051
Insulin Therapy, n (%)	164 (16.3)	82 (15.2)	82 (17.7)		0.275
AADs, n (%)	75 (7.4)	46 (8.5)	29 (6.3)		0.178
Statins, n (%)	224 (22.3	129 (23.8)	95 (20.5)		0.206
Dabigatran, n (%)	148 (14.7)	90 (16.6)	58 (12.5)		0.067
Rivaroxaban, n (%)	402 (40.0)	214 (39.6)	188 (40.6)		0.735
Apixaban, n (%)	252 (25.0)	144 (26.6)	108 (23.3)		0.231
Edoxaban, n (%)	202 (20.1)	93 (17.1)	109 (23.5)		**0.012**
Clinical, hemodynamic and laboratory parameters					
BMI, kg/m^2	23.3 ± 3.1	23.6 ± 3.2	23.0 ± 3.0		** < 0.0001**
SBP, mmHg	136.4 ± 10.6	136.4 ± 10.4	136.3 ± 10.8		0.957
DBP, mmHg	86.5 ± 24.5	85.8 ± 7.9	87.3 ± 35.1		0.324
Heart rate, bfm	82.4 ± 7.5	82.6 ± 7.4	82.1 ± 7.7		0.254
Total Cholesterol, mg/dl	139 ± 31.4	137.3 ± 30.9	141 ± 31.8		0.065
HDL, mg/dl	48.7 ± 8.3	48.7 ± 7.9	48.6 ± 8.7		0.773
LDL, mg/dl	81.4 ± 22.5	81.3 ± 21.9	81.5 ± 23.1		0.884
Triglycerides, mg/dl	139.2 ± 43.1	139.2 ± 43.2	139.3 ± 42.9		0.978
Creatinine, mg/dl	1.1 ± 0.5	1.1 ± 0.6	1.07 ± 0.3		0.534
ClCr (Cockcroft-Gault)	44.6 ± 16.6	48.6 ± 17.9	40 ± 13.6		** < 0.0001**
eGFR, ml/min/1.73 m2	60.3 ± 17.7	62.1 ± 18	58.1 ± 17		** < 0.0001**
Hb, g/dl	11.5 ± 1.7	11.7 ± 1.9	11.4 ± 1.5		**0.008**
PLTs × 10^3/uL	244.1 ± 84.5	239.5 ± 84.5	249.3.4 ± 84.4		0.063
Serum Albumin, g/dl	3.5 ± 0.7	3.5 ± 0.6	3.5 ± 0.7		0.672
GOT/AST, U/ml	32.6 ± 46.3	30.7 ± 42.3	34.7 ± 50.7		0.176
GPT/ALT, U/ml	29.9 ± 44.7	28.5 ± 41.2	31.4 ± 48.4		0.303
Total bilirubin, mg/dl	1.2 ± 3.7	1.0 ± 1.4	1.4 ± 5.2		0.081
Direct bilirubin, mg/dl	0.4 ± 1.7	0.3 ± 1.3	0.5 ± 2.0		0.188
ALP, U/L	104.5 ± 57.7	101.3 ± 54	108.2 ± 61.6		0.058
GGT, U/L	31.9 ± 31.3	33.3 ± 35.5	30.2 ± 25.5		0.125
Glycaemia, mg/dl	102.4 ± 35.6	101.0 ± 36.9	104.2 ± 34.1		0.150
Uric acid, mg/dl	6.1 ± 3.3	6.1 ± 3.9	6.1 ± 2.3		0.880
MMSE, pt	23.1 ± 6.0	25.9 ± 4.5	19.8 ± 5.9		** < 0.0001**
MoCA, pt	25 ± 2.9	26.2 ± 2.5	23.6 ± 2.7		** < 0.0001**
GDS, pt	5 ± 4.3	2.9 ± 3.0	7.3 ± 4.3		** < 0.0001**
ADL, pt	4.1 ± 1.7	5.3 ± 1.1	2.8 ± 1.4		** < 0.0001**
IADL, pt	5.3 ± 2.4	6.8 ± 1.6	3.4 ± 1.8		** < 0.0001**
SPPB, pt	7.4 ± 3.7	10.4 ± 1.6	3.9 ± 2.2		** < 0.0001**

Bold values indicate statistically significant p values

*T2DM* type 2 Diabetes mellitus, *NAFLD* Non-alcoholic fatty liver disease, *RF/COPD* respiratory failure/Chronic Obstructive pulmonary Disease, *IHD* Ischemic Heart Disease, *HF* Heart Failure, *VKAs* Vitamin K antagonist, *DOACs* Direct oral anticoagulants, PLTs platelets, *RAASi* Renin–angiotensin–aldosterone system inhibitors, CCBs Calcium channel blockers, *OADs* Oral anti-diabetic drugs, *AADs* Anti-arrhythmic drugs, *BMI* Body mass index, SBP Systolic Blood pressure, DBP Diastolic Blood pressure, *HDL* High Density Lipoprotein, *LDL* Low Density Lipoprotein, *CrCl* Creatinine clearance, *e-GFR* estimate Glomerular Filtration Rate, *Hb* Hemoglobin, GOT/AST Glutamic Oxaloacetic Acid/Aspartate Aminotransferase, *GTP/ALT* Glutamic Pyruvic/Alanine Aminotransferase, *ALP* Alkaline phosphatase, *GGT* Gamma Glutamyl Transferase, *HOMA* Homeostasis Model Assessment, *MMSE* Mini mental state examination, *MoCA* Montreal Cognitive Assessment, *GDS* Geriatric Depression Scale, *ADL* Activities of Daily Living, *IADL* Instrumental Activities of Daily Living, *SPPB* Short Performance physical battery

Anthropometric, hemodynamic, and biochemical characteristics of the entire population stratified according to the SPPB score were also shown in Table 1. The group with pathological SPPB had worse values of BMI (23.6 ± 3.2 vs 23.0 ± 3.0 kg/m^2^; p < 0.0001), eGFR (62.1 ± 18.0 vs 58.1 ± 17.0 ml/min/1.73 m^2^; p < 0.0001), CrCl (48.6 ± 17.9 vs 40 ± 13.6; p < 0.0001) and hemoglobin (11.7 ± 1.9 vs 11.4 ± 1.5 g/dl; p = 0.008). than the group with SPPB ≥ 8 pt. No statistically significant differences were found for SBP, DBP, total cholesterol, HDL, LDL, or triglycerides. Of interest, patients with a worse SPPB value had worse cognitive function, more depressive symptoms and more functional limitations detected at CGA (see Table 1). Table 2 shows the major cardiovascular events (CVEs) in the study population. A total of 250 CVEs were observed (7.7 events/100 patient-years), 66 in the group with SPPB ≥ 8 points (3.8 events/100 patient-years) and 184 in the group with SPPB < 8 points (12.4 events/100 patient-years), p < 0.0001. Of the total CVES, 64 were nonfatal strokes (NFS) (2.0 events/100 patients-years), with 16 (0.9 events/100 patients-years) in the first group and 48 (3.2 events/100 patients-years) in the second, p < 0.0001; 85 (2.6 events/100 patients-years) were nonfatal coronary events (NCE), with 23 events (1.3 events/100 patients-years) in the first group and 62 in the second (4.3 events/100 patients-years), p < 0.0001. Regarding cardiovascular death, 101 events were recorded in the study population (3.1 events/100 patient-years), 35 events (2.0 events/100 patient-years) were recorded in the group with SPPB ≥ 8 points, and 66 (4.4 events/100 patient-years) in the pathological SPPB group, p < 0.0001. Also non-cardiovascular mortality: 219 events (6.8 events/100 patient-years), was higher in the pathological SPPB group 148 (9.9 events/100 patient-years) than other group 71 events (4.1 events/100 patient-years) p < 0.0001, respectively. Finally, the total number of deaths were 320 (9.9 events/100 patient-years), 106 (6.1 events/100 patient-years) in the pathological SPPB group and 214 (14.4 events/100 patient-years) in the second group, p < 0.0001. From the results of the Cox linear regression analysis on the incidence of CVEs, (Supplementary Table 1), a statistically significant association emerged for ischemic heart disease, statin treatment, male sex, the presence of Non-alcoholic fatty liver disease (NAFLD), VKA use before DOACs treatment, antiplatelet therapy before DOACs treatment, treatment with renin-angiotensin- aldosterone system inhibitors (RAASi), pathological GDS, and pathological SPPB (p < 0.0001).

**Table 2:** Cardiovascular events in the study population

	All population (n.1004)	SPPB ≥ 8 (n. 541)	SPPB < 8 (n. 463)	p*
CVEs, n (%)	250 (7.7)	66 (3.8)	184 (12.4)	** < 0.0001**
Nonfatal Stroke, n (%)	64 (2.0)	16 (0.9)	48 (3.2)	** < 0.0001**
Nonfatal Coronary events, n (%)	85 (2.6)	23 (1.3)	62 (4.3)	** < 0.0001**
Cardiovascular mortality, n (%)	101 (3.1)	35 (2.0)	66 (4.4)	** < 0.0001**
Non-cardiovascular mortality, n (%)	219 (6.8)	71 (4.1)	148 (9.9)	** < 0.0001**
Total mortality, n (%)	320 (9.9)	106 (6.1)	214 (14.4)	** < 0.0001**

Bold values indicate statistically significant p values

^*^performed with chi square test

Data described as number of patients (number of events per 100 patient-year)

*CVEs* Cardiovascular events, *SPPB* Short physical performance battery

**Fig. 2:**
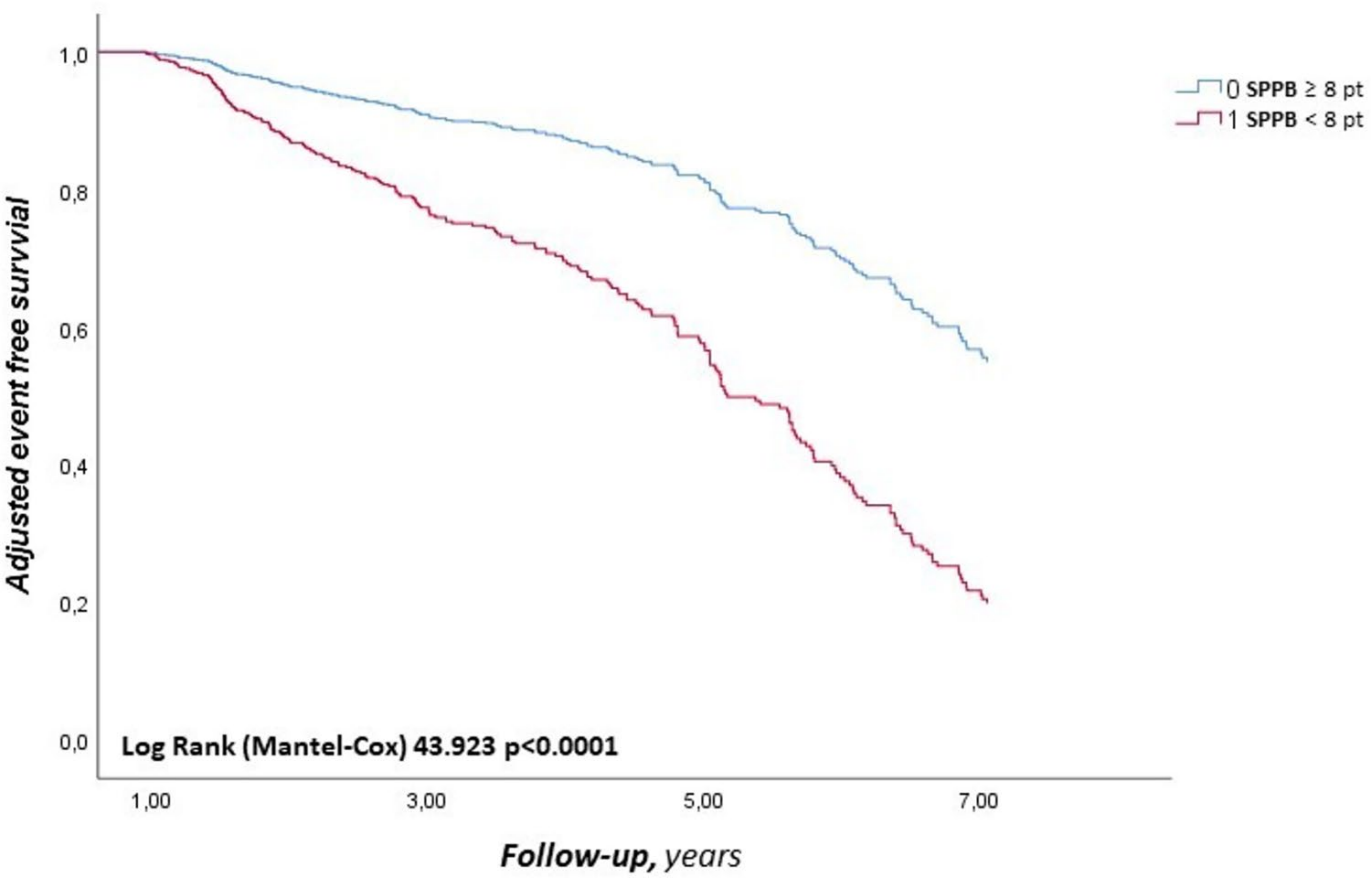
Kaplan–Meier survival curve for the two different groups stratified according to SPPB value. *SPPB* Short physical performance battery, *CVEs* cardiovascular events

Variables that were statistically significantly associated with the onset of CVEs in the univariate Cox analysis were included in a multivariate stepwise analysis model to define independent predictors of CVEs (Table 3). Specifically, statin treatment was associated with a 41% reduction of risk of CVEs (HR 0.59, CI 0.44–0.79; p < 0.0001), male sex with a reduced risk of CVEs by 34% (HR 0.66, CI 0.49–0.88; p = 0.010), and RAASi treatment was associated with a 33% reduction of risk of CVEs (HR 0.67, CI 0.51–0.89; p = 0.006). The presence of a pathological SPPB score (< 8 points) was associated with an increased risk of CVEs by 2.78 times compared to the non-pathological SPPB group (HR 2.78, CI 2.04–3.81; p < 0.0001), while a pathological GDS score with an increased risk by 54% (HR 1.54, CI 1.16–2.03; p = 0.002); VKAs treatment before DOACs treatment was associated with an increased risk of CVEs by 35% (HR 1.35, CI 1.04–1.74; p = 0.022). The AUC was used to evaluate the accuracy of SPPB as a predictive value for the onset of CVEs as a dichotomous variable above and below the median. Figure 1 shows the ROC curve of SPPB as a dichotomous variable: AUC 0.680; (standard error 0.019; 95% CI 0.643–0.718; p < 0.0001). Figure 2 shows the survival curves for the two different subgroups.

**Table 3:** Multivariate Cox linear regression on CVES incidences

	HR	CI 95%	p
Statins, yes/no	0.59	0.44–0.79	** < 0.0001**
Male sex, yes/no	0.66	0.49–0.88	**0.010**
RAASi, yes/no	0.67	0.51–0.89	**0.006**
Pathological SPPB, yes/no	2.78	2.04–3.81	** < 0.0001**
Pathological GDS, yes/no	1.54	0.16–2.03	**0.002**
VKAs before DOACs, yes/no	1.35	1.04–1.74	**0.022**

Bold values indicate statistically significant p values

*CVEs* Cardiovascular events, *RAASi* Renin–angiotensin–aldosterone system inhibitors, *SPPB* Short physical performance battery, *GDS* Geriatric Depression Scale, *VKAs* Vitamin K antagonist, *DOACs* Direct oral anticoagulants

## Discussion

Our study including elderly patients with AF demonstrates a high risk of worse clinical outcomes in patients hospitalized and with a reduced functional mobility.

In particular, from the Cox multivariate regression model, it emerged that having an SPPB < 8 points is associated with a 2.78-fold higher risk of CVEs compared to an SPPB ≥ 8 points. The analysis of the processed ROC curve and measurement of relative AUC also demonstrated the accuracy of SPPB pathological value as predictor of CVEs occurrence. These results are clinically relevant because functional limitation is frequent in elderly patients. Of particular interest, our study shows that treatment with statins, male gender, and RAASi treatment were associated with a reduced risk of CVEs incidence by 41%, 34%, and 33%, respectively; while pathological GDS and treatment with VKAs prior to DOACs therapy were associated with an increased risk of major cardiovascular events by 54% and 35%, respectively. Functional limitation, therefore, associated with decreased exercise tolerance, seems to increase the risk of nonfatal stroke, nonfatal acute coronary events, and both cardiovascular and non-cardiovascular mortality. This is relevant because AF is often associated with other risk factors and clinical conditions that can contribute to functional limitation and worsen prognosis, such as advanced age, comorbidities, frailty, and lifestyle; these factors increase the risk of adverse events in patients and negatively influence prognosis. To the best of our knowledge, there are no studies that have evaluated SPPB as a prognostic factor associated with the risk of CVEs in hospitalized elderly patients with NVAF receiving DOACs therapy. However, several studies have been conducted on the elderly population and have correlated pathological SPPB with several endpoints. A prospective cohort study evaluated the association between SPPB and CoI in elderly patients and found that decreased physical performance, assessed as SPPB < 9 points, was independently associated with the risk of CoI (HR 2.222, 95% CI 1.047–4.716, p = 0.038) [19]. Similar results were found in our study, as the group with SPPB < 8 points showed significantly lower MMSE and MoCA values compared to the group with SPPB ≥ 8 points [MoCA ≤ 26 points (23.6 ± 2.7 vs. 26.2 ± 2.5, p < 0.0001) and MMSE ≤ 24 points (19.8 ± 5.9 vs. 25.9 ± 4.5, p < 0.0001)]. This can be explained by the presence of common risk factors for both conditions, such as advanced age, comorbidities, and inflammatory status. Literature has revealed data on the association between AF and functional limitation in the elderly, independent of comorbidities and frailty markers. Indeed, a prospective cohort study evaluated the association between AF and the risk of reduced functional ability, assessed with ADL, in elderly patients, independent of the presence of stroke, heart failure, and other comorbidities. The same study also discussed how AF, independently of stroke, can exert degenerative effects on the Central nervous system through hypoperfusion and transient ischemic attacks (TIAs) [20]. Silent strokes and white matter disease are associated with gait and balance abnormalities [21], disability, and a higher risk of falls. Several cross-sectional studies have observed associations between AF and the presence of white matter abnormalities or TIAs [22]. In another study, physical function was assessed with SPPB and its potential association with a composite endpoint consisting of ischemic heart disease, stroke, and heart failure, as well as with individual cardiovascular endpoints, was assessed. It was found that reduced physical performance was an independent predictor of the evaluated cardiovascular outcomes, both as a composite endpoint and individually. This study included 5,570 participants with a mean age of 75 ± 5 years, of whom 58% were female, regardless of prior CVD history. In the Atherosclerosis Risk in Communities (ARIC) study, the population was divided into three groups according to SPPB value: low (0–6 points), intermediate (7–9 points), and high (10–12 points). During a median follow-up of 7 years (IQR range 5.3–7.8 years), 930 composite cardiovascular events occurred. Multivariate Cox regression model showed that the risk of achieving a composite endpoint was 47% higher in the low SPPB group compared to the high SPPB group (HR 1.47, 95% CI 1.20–1.79), and 25% in the intermediate group compared to the low SPPB group, a result that persisted even after adjusting for potential confounders. This study confirms the important prognostic role of functional limitation, assessed with SPPB, as an independent risk factor for composite cardiovascular endpoints in elderly population, regardless of other traditional risk factors [23]. However, the average age of the population in this study was lower than in our study (75 vs. 84 years), and they were outpatients, not all suffering from AF and with a lower burden of comorbidities than in our study.

Of interest, another study showed that after a diagnosis of AF, regardless the presence of stroke, there is a loss of functional autonomy with an increased likelihood of needing assistance, increasing the risk of disability [24].

Our work is also in agreement with previous studies that have demonstrated how reduced functional ability, assessed with ADL < 3, in elderly patients diagnosed with AF and treated with DOACs, is pathologically associated with the risk of CVEs. Therefore, in the hospitalized elderly population with AF, it seems important to evaluate not only the classic risk factors but also the possible presence of functional limitation assessed using SPPB. Indeed, in our work, it appears that a pathological SPPB increases the risk of CVEs by 2.78 times during a follow-up of 3.2 years. Additionally, an increased risk is associated with a treatment with VKAs before DOACs treatment, and the presence of a pathological GDS; while statin treatment, male gender, and RAASi treatment were found to be protective [25]. Furthermore, our study shows that patients with an SPPB score ≥ 8pt exhibited better scores on geriatric scales than the SPPB group < 8 pt, such as GDS 2.9 ± 3. 0 vs 7.3 ± 4.3 pt, p < 0.0001; ADL 5.3 ± 1.1 vs 2.8 ± 1.4 pt, p < 0.0001; and IADL 6.8 ± 1.6 vs 3.4 ± 1.8 pt, p < 0.0001; demonstrating the importance of this test in the elderly population. This study has several limitations; it is an observational study and not a randomized controlled clinical trial. Furthermore, the ratio between the two groups is not 1:1, which represents an intrinsic limitation of the study. Finally, we cannot exclude residual confounding factors not addressed in this study. However, this study has several strengths: it is a study with a large sample size consisting of elderly patients with several comorbidities often excluded from randomized clinical trials. Finally, the long follow-up represents another strength of the study.

## Conclusions

In conclusion, the study demonstrated that in hospitalized elderly patients with NVAF treated with DOACs, functional limitation, assessed by a score lower than 8 on the SPPB test, was associated with an increased risk of CVEs by 2.78-fold, thereby confirming the importance of functional limitation in cardiovascular prognosis. Additionally, an increased risk is associated with a treatment with VKAs prior to DOACs therapy, and a pathological GDS, while statin treatment, male gender, and RAASi therapy were found to be protective.

## Supplementary Information

Below is the link to the electronic supplementary material.

Supplementary file1 (DOCX 16 KB)

## Data Availability

Data available on request. The data underlying this article will be shared on reasonable request to the corresponding author.
