# Supplementary material for: Prognostic role of short physical performance battery in elderly hospitalized atrial fibrillation patients
Source: Intern Emerg Med. 2025 May 16;20(5):1389–97. doi: 10.1007/s11739-025-03958-8 (PMC12331861; doi:10.1007/s11739-025-03958-8)
Supplement: Supplementary file 1 — Supplementary file1 (DOCX 16 KB) [file 11739_2025_3958_MOESM1_ESM.docx]

## Table 4. Cox linear regression on MACE incidences

|  | **HR** | **CI 95%** | **p** |
| --- | --- | --- | --- |
| **Male sex,** yes*/no* | 0.62 | 0.43-0.91 | **0.014** |
| ***Age*,** *10 years* | 0.94 | 0.72-1.22 | 0.622 |
| **Smoke,** yes*/no* | 1.19 | 0.85-1.68 | 0.316 |
| **Alcool,** *yes/no* | 1.25 | 0.75-2.06 | 0.388 |
| **Arterial Hypertension,** *yes/no* | 1.10 | 0.61-1.99 | 0.736 |
| **T2DM,** *yes/no* | 1.07 | 0.69-1.64 | 0.753 |
| **NAFLD,** *yes/no* | 1.95 | 1.05-3.62 | **0.034** |
| **RF/COPD,** *yes/no* | 1.15 | 0.86-1.54 | 0.353 |
| **IHD,** *yes/no* | 1.58 | 0.95-2.64 | 0.076 |
| **HF,** *yes/no* | 1.05 | 0.77-1.43 | 0.744 |
| **CHA2DS2VASc,** *1 pt* | 1.05 | 0.90-1.23 | 0.507 |
| **BMI,** *1 Kg/m2* | 0.98 | 0.93-1.03 | 0.466 |
| **SBP,** *1 mmHg* | 1.00 | 0.98-1.03 | 0.478 |
| **DBP,** *1 mmHg* | 0.99 | 0.98-1.01 | 0.493 |
| **VKAs pre-DOACs,** yes*/no* | 1.34 | 1.01-1.77 | **0.040** |
| **Anti-PLTs pre-DOACs,** *yes/no* | 0.68 | 0.44-1.05 | 0.084 |
| **Anti-PLTs,** *yes/no* | 1.10 | 0.54-2.28 | 0.778 |
| **Nitrates,** *yes/no* | 0.79 | 0.43-1.47 | 0.463 |
| **RAASi,** yes*/no* | 0.62 | 0.44-0.87 | **0.006** |
| **β-blocker** yes*/no* | 0.81 | 0.57-1.15 | 0.247 |
| **Digitalis,** yes*/no* | 0.84 | 0.55-1.30 | 0.441 |
| **OADs,** *yes/no* | 0.79 | 0.50-1.25 | 0.326 |
| **AADs,** *yes/no* | 0.98 | 0.59-1.63 | 0.947 |
| **Statins,** yes/no | 0.55 | 0.39-0.78 | **0.001** |
| **LDL,** *1 mg/dl* | 1.00 | 0.99-1.00 | 0.576 |
| **Triglycerides,,** *1 mg/dl* | 0.99 | 0.99-1.00 | 0.453 |
| **CrCl,** *1 ml/min* | 1.00 | 0.99-1.02 | 0.554 |
| **Hb,** *1 g/dl* | 1.06 | 0.98-1.14 | 0.140 |
| **PLTs,** *1000/uL* | 1.00 | 0.99-1.00 | 0.423 |
| **Serum Albumin,** *1 g/dl* | 1.02 | 0.80-1.29 | 0.853 |
| **GOT/AST,** *1UI/L* | 1.00 | 0.99-1.00 | 0.997 |
| **GPT/ALT,** *1UI/L* | 0.99 | 0.98-1.00 | 0.123 |
| **Total Bilirubin ,** *1 mg/dl* | 0.99 | 0.97-1.02 | 0.833 |
| **ALP,** *1 UI/L* | 1.00 | 0.99-1.00 | 0.239 |
| **GTT,** *1 UI/L* | 0.99 | 0.99-1.00 | 0.528 |
| **Uric Acid,** *1mg/dl* | 0.98 | 0.92-1.05 | 0.613 |
| **Pathological MoCA,** *yes/no* | 1.12 | 0.79-1.57 | 0.527 |
| **Pathological GDS,** *yes/no* | 1.57 | 1.13-2.17 | **0.006** |
| **Pathological SPPB,** *yes/no* | 3.28 | 2.26-4.76 | **<0.0001** |

**Abbreviations:** T2DM, type 2 Diabetes mellitus; NAFLD, Non-alcoholic fatty liver disease; RF/COPD, respiratory failure/Chronic Obstructive pulmonary Disease; IHD, Ischemic Heart Disease; HF, Heart Failure; VKAs, Vitamin K antagonist; DOACs, Direct oral anticoagulants; PLTs, platelets; RAASi, Renin-angiotensin-aldosterone system inhibitors; OADs, Oral anti-diabetic drugs; AADs, Anti-arrhythmic drugs; HOMA, Homeostasis Model Assessment; BMI, Body mass index; SBP, Systolic Blood pressure; DBP, Diastolic Blood pressure; HDL, High Density Lipoprotein; LDL, Low Density Lipoprotein; CrCl, Creatinine clearance; e-GFR, estimate Glomerular Filtration Rate; Hb, Hemoglobin; GOT/AST, Glutamic Oxaloacetic Acid/Aspartate Aminotransferase; GTP/ALT, Glutamic Pyruvic/Alanine Aminotransferase; ALP, Alkaline phosphatase; GGT, Gamma Glutamyl Transferase; MoCA, Montreal Cognitive Assessment; GDS, Geriatric Depression Scale; SPPB, Short Performance physical battery.
